# Telemonitoring in the Covid-19 Era: The Tuscany Region Experience

**DOI:** 10.3390/healthcare9050516

**Published:** 2021-04-29

**Authors:** Silvia Panicacci, Massimiliano Donati, Alberto Lubrano, Annamaria Vianello, Alessio Ruiu, Luca Melani, Antonella Tomei, Luca Fanucci

**Affiliations:** 1 Department of Information Engineering, University of Pisa, Via G. Caruso 16, 56122 Pisa, Italy; massimiliano.donati@unipi.it (M.D.); alberto.lubrano@dii.unipi.it (A.L.); annamariavianello@med.unipi.it (A.V.); luca.fanucci@unipi.it (L.F.); 2 IngeniArs S.r.l., 56121 Pisa, Italy; alessio.ruiu@ingeniars.com; 3 AFT Pisa 2, Azienda USL Toscana Nord-Ovest, 56121 Pisa, Italy; lucamelani@libero.it; 4 Dipartimento Sanità Territoriale, Azienda USL Toscana Nord-Ovest, 56121 Pisa, Italy; antonella.tomei@uslnordovest.toscana.it

**Keywords:** Covid-19, telemedicine, remote monitoring, sensors, treatment plan, cloud platform

## Abstract

Covid-19 has brought many difficulties in the management of infected and high-risk patients. Telemedicine platforms can really help in this situation, since they allow remotely monitoring Covid-19 patients, reducing the risk for the doctors, without decreasing the efficiency of the therapies and while alleviating patients’ mental issues. In this paper, we present the entire architecture and the experience of using the Tel.Te.Covid19 telemedicine platform. Projected for the treatment of chronic diseases, it has been technologically updated for the management of Covid-19 patients with the support of a group of doctors in the territory when the pandemic arrived, introducing new sensors and functionalities (e.g., the familiar use and video calls). In Tuscany (Central Italy), during the first wave of outbreak, a model for enrolling patients was created and tested. Because of the positive results, the latter has been then adopted in the second current wave. The Tel.Te.Covid19 platform has been used by 40 among general practitioners and doctors of continuity care and about 180 symptomatic patients since March 2020. Both patients and doctors have good opinion of the platform, and no hospitalisations or deaths occurred for the monitored patients, reducing also the impact on the National Healthcare System.

## 1. Introduction

The SARS-CoV-2 infection, responsible for the Covid-19 clinical syndrome, originated in Wuhan in China and then spread all over the world, is characterised by a very broad clinical spectrum, ranging from almost asymptomatic forms, to fear-symptomatic forms, to patterns with bilateral and systemic lung involvement, to dramatic and lethal situations [1,2]. As expected, older people with comorbidities have the highest risk of a poor prognosis and need intensive monitoring to prevent rapid clinical deterioration. Multiple studies have confirmed that age, cardiovascular diseases (e.g., coronary artery disease, heart failure, cardiac arrhythmia and hypertension) and diabetes are the main risk factors [3,4]. However, there is evidence of pneumonia related to Covid-19 also in people aged around 50 years.

Because of their high risk, frail patients suffering from chronic conditions are confined to their home, allowing hospitalisation only in emergencies, due to the high risk of infections in hospitals, clinics and doctors’ offices. Physical estrangement and social isolation cause these frail patients to feel abandoned, anxious and depressed, sometimes with consequences worse than the disease itself [5]. Covid-19 could also be associated with neurological manifestations [6] and could improve the risk for neuro-degenerative diseases [7].

In this pandemic scenario, technology can play a key role [8]. First of all, there is a need for intensive remote monitoring of the vital parameters of infected patients with pulmonary involvement and high-risk patients, allowing early diagnosis [9]. Secondly, visits to infected patients by general practitioners (GPs) should be reduced, to decrease the risk of infection for GPs themselves and to allow them time to follow up other chronic conditions. In addition, an informatics platform where GPs, specialists and primary care physicians interact together could be very important in this situation. Finally, patients need something to alleviate the mental health problems caused by physical distancing and social isolation, while also trying to prevent the development of neuro-degenerative diseases [10].

Telemedicine can really help to address these issues, as it concerns the remote monitoring of vital parameters. Thus, it could be useful in minimising the spread of the virus, reducing contact between doctors and patients, better allocating healthcare resources, reducing anxiety and depression in patients through continued home monitoring [11,12,13,14] and fighting neuro-degenerative diseases [15].

Before the pandemic, a telemedicine platform was used in Tuscany (Central Italy) to monitor chronic patients (e.g., with heart failure) with positive results and feedbacks [16,17]. With the arrival of the outbreak, after some requests from the GPs working on the territory, the system was technologically updated for the Covid-19 emergency, responding to some specific situations. Then, it was used to remotely monitor infected patients from March 2020.

This paper presents Tel.Te.Covid19, the telemedicine platform adapted and used in Tuscany for the management of Covid-19 patients at home. In particular, the paper describes the architecture of the system and the upgrades made for the pandemic and the experience of using this platform in Tuscany. Moreover, it illustrates a model for enrolling patients in the telemedicine program, based on the timing of symptoms. This model has been created by some doctors during the first wave of the outbreak and has also been adopted during the second wave, achieving no hospitalisations and deaths.

After this Introduction, Section 2 reports the technical description of the Tel.Te.Covid19 platform, highlighting the advancements with respect to the version for chronic patients. Results on the Tuscan territory are presented and discussed in Section 3, while conclusions are drawn in Section 4.

## 2. Material and Methods

In the Local Health Unit of North-West Tuscany, a project for the territorial telemonitoring of chronic patients (Tel.Te.C.) has been running since 2017. Tel.Te.C. is a telemedicine system, composed of a mobile application for in-house collection of vital parameters and a cloud platform which allows the GPs to monitor them remotely [18]. 20 GPs were involved in the project in February 2020. It consisted in enrolling heart failure patients and remotely monitoring heart rate (HR), oxygen saturation (SpO${}_{2}$), blood pressure and weight according to a personalised care plan decided and customisable by the doctor, specialist and other members of the care team [19].

On 25 February 2020, the first Tuscan Covid-19 positive patient was detected, a few days after the arrival of the virus in the North of Italy [20]. The GPs already involved in Tel.Te.C. suggested updating the existing telemedicine platform to monitor Covid-19 patients, in order to minimise contacts between patients and doctors, reduce the number of hospital admissions and intensive and sub-intensive care, thanks to a continuous monitoring similar to a hospitalisation, and provide early diagnosis especially for high-risk patients, identifying the infection as soon as possible. On this basis, we developed Tel.Te.Covid19, adapting the previous version of the system to the current outbreak.

**Figure 1:**
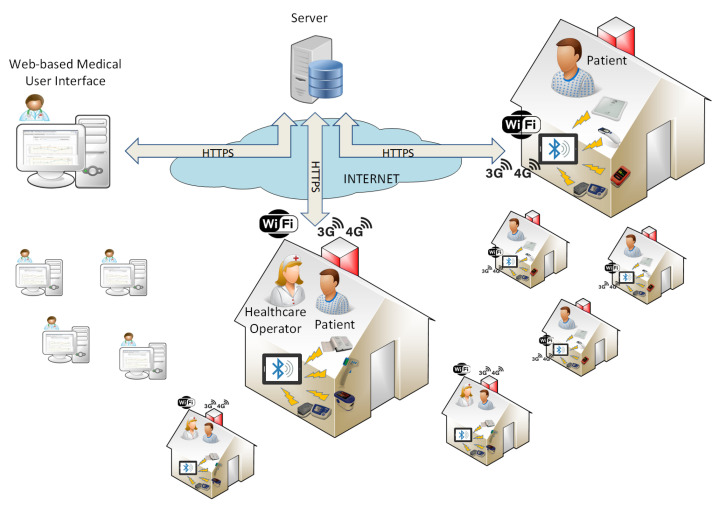
Architecture of the Tel.Te.Covid19 telemedicine platform.

More in detail, the system is composed of three modules [21], as shown in Figure 1:

- the cloud platform, multi-access and multi-profile, delivered on the web. It features a graphical user interface (GUI), which allows doctors and specialists who are members of the care team (after the log in operation) to enrol patients, customise the monitoring plan (i.e., the number and type of measurements) and the thresholds which generate events and notifications (e.g., SpO${}_{2}$ lower than 95% generates a serious event for patient A and a minor event for patient B), and visualise the trend of the collected measurements;
- the home kit, delivered to a single patient. It is composed of a set of Bluetooth sensors and an Android tablet with an application installed. The latter notifies through audio/video reminders and guides the patient in performing self-measurements of vital parameters by means of the sensors, according to the personalised measurement plan, sent from the cloud to the application. Moreover, it is in charge of transmitting the collected values to the web platform, to make them available for the care team. The patient can also use the application to make extra measurements;
- the professional kit, for nurses and healthcare operators. It was designed to support professionals during home visits to the patients in charge or to manage several patients within the same healthcare facility. It is composed of a set of Bluetooth sensors, which are usually more professional than those in the home kit, and an Android tablet with the professional application installed.

This patient-centric platform is based on a client-server architecture, where the server is the cloud platform, always online to receive data and update the vital parameters graphs in real time for the members of the care team, while the tablets are the multiple clients. They connect to the server via Internet (i.e., 3G, 4G, or WiFi). Authentication to the server and HTTPS ensure the security and privacy of sensible data, according to the current regulations (i.e., GDPR). Finally, the system is fault-tolerant: the mobile application shows if the required measurement has been correctly collected via Bluetooth through the sensor and, in case of network outages (if the tablet cannot send data to the server), it saves data locally and sends them as soon as connectivity becomes available again.

At the beginning of the pandemic, the idea was to deliver home kits (sensors and tablets) to infected patients, to continuously remotely monitor their vital parameters at home.

With respect to the version for chronic patients, a thermometer has been integrated into the system, since temperature is one of the most important parameters to control Covid-19 patients. The Bluetooth sensors included in the home kit are shown in Table 1, and allow the monitoring of body temperature, SpO${}_{2}$, finger HR, blood pressure and weight. Moreover, the possibility to manually enter the measurements in the mobile application was given, enabling also the use of sensors that people have usually at home, such as thermometers. However, when possible, integrated Bluetooth sensors are preferable, because they avoid errors due to human intervention.

To increase flexibility and scalability during the emergency, we transformed the home kit from personal to familiar, modifying the cloud platform and giving patients access to the web. In fact, at the beginning of the outbreak, it was clear that if a person is Covid-19 positive, it is very probable that their cohabitants are also infected. Then, we provided the functionality of registering in the web platform to patients. In this way, they have become able to log and manually enter the required measurements directly in the web platform (bypassing the mobile application): the tablet remains personal, while the sensors can be shared by all family members. Therefore, one home kit can be used by all the people living in the same house, at the cost that only one patient has the functionality of audio/video reminders to respect the personalised care plan. The possibility to autonomously register and enter measurements in the web platform also allows people without the home kit to be continuously monitored by the doctors: only a thermometer and a pulsoximeter are required at home to collect the vital parameters and autonomously remember when to take measurements.

We have added also the video call to the platform, in order to create a direct contact between the doctor and the patient in-app. The call is one-way: doctors can call one of their patients, while patients cannot call the members of the care team. In fact, this video call should be intended as a remote visit, which the doctor can use for checks or to control some vital parameters, avoiding a home visit whenever possible. It is not an emergency line where patients can call doctors at any time, but it is a care tool, which can also alleviate the sense of abandonment resulting from social distancing and isolation, since patients are aware that doctors can video call them in case there is something strange. The process to video call is simple: when a doctor wants to call one of his/her patients, he/she has to log in the professional mobile application and select the patient in the list. The patient receives the incoming call from the app on the tablet. During the video call, both doctors and patients can switch audio and video on/off and change the camera (from front to back and vice versa). The video calls are managed entirely within the mobile applications, exploiting the QuickBlox platform [26]. It provides SDKs and a server, which acts as an intermediary to exchange the information needed to set up the call. Once the video call has been set up, it then proceeds via a direct connection between the two tablets. Therefore, patients’ privacy is protected: the information passing through the QuickBlox servers relates exclusively to the calls and does not contain any data on doctors or patients.

The very complicated situation required acting quickly without losing time, also in enrolling patients, since the timing of remote monitoring is crucial to avoid hospital admissions. For this reason, to facilitate and speed up patient enrolment, we have provided the web platform with two standard monitoring plans, making recruitment automatic. The two monitoring plans, which differ in the number of measurements per day, are the following:

- intensive monitoring plan: it requires 4 measurements per day of body temperature, SpO${}_{2}$ and HR and 2 measurements per day of blood pressure and weight;
- moderate monitoring plan: it requires 2 measurements per day of body temperature, SpO${}_{2}$ and HR and 1 measurement per day of blood pressure and weight.

However, also these plans can be customised by the doctors, if the patient requires a different treatment and type of monitoring.

## 3. Results and Discussion

The Tel.Te.Covid19 telemedicine system, with sensors, mobile applications and cloud platform, makes it possible to visualise in real time the trend of patient parameters and, above all, the variations of these vital parameters after the introduction of the anti-Covid therapy. Patients clinical data are shared among all members of the care team, who can visualise and analyse them on the web. Therefore, patients can be treated at home, even when their parameters get worse, thanks to the continuous monitoring similar to that provided during a hospital admission, also making it possible to react quickly by changing the drug therapy.

The functionalities of autonomously registering in the web platform and manually entering the collected measurements allow increasing the scalability of the entire system. In fact, the GPs can enrol and monitor more patients than before: (i) patients with the complete home kit (Bluetooth sensors and tablet), (ii) cohabitants of enrolled patients, who use Bluetooth sensors to collect their vital parameters and manually enter the values in the web, and (iii) patients with their own sensors, who exploit only the web platform. In this way, not all patients need to have the Tel.Te.Covid19 home kit to be monitored, leading to a reduction in the number of home kits to provide and lowering costs.

Via the graphs on the web platform, it is also possible to note that the sensors provided in the home kit have shown great precision in the measurements, increasing the reliability of the entire system.

Considering the experience on the territory, in the period March–June 2020, corresponding to the first wave of the Covid-19 pandemic, the knowledge of the disease was quite deficient. The Tuscan doctors already involved in Tel.Te.C. saw the potentiality of Tel.Te.Covid19 for the management of infected patients and tried to monitor them according to the severity of their disease. They decided to use the telemedicine platform in different ways depending on the patients’ conditions, as explained below:

- the first stage is characterised by flu-like symptoms, such as asthenia, fatigue, myalgia, headache, fever, dry cough, nausea and diarrhoea. In this case, patients should monitor body temperature, SpO${}_{2}$ and HR twice per day (in the morning and in the evening) and blood pressure and weight once per day (moderate monitoring plan). For this type of monitoring, patients could exploit the new functionality of registering in the web platform and manually enter the vital parameters, using the sensors on their own. In this way, doctors could monitor a possible worsening and identify the exact moment when the disease was ending up in the second stage, acting promptly;
- the second stage is represented by hyper inflammatory activation, pneumonia and usually multi-organ involvement, with different spectrum of clinical manifestation and high risk of hospitalisation. If the GP confirmed stage two entry, i.e., signs of pleuro-pulmonary involvement, with a home visit, patients were given the home kit, complete with Bluetooth sensors and a tablet, and the intensive monitoring plan together with an intensive drug therapy was started, according to the current guidelines for the treatment of Covid-19 patients with lung involvement. In fact, each time a patient progresses to the second stage of Covid-19, intensive home monitoring represents a real alternative to in-hospital clinical monitoring.

The enrolment of patients was extremely rapid, thanks to the standard monitoring plans provided by the platform, which allowed patients to be automatically entered into the program without having to choose the number of measurements for each parameter each time.

With the first patients enrolled in Tel.Te.Covid19 according to this protocol, via the vital parameters graph in the web platform, the doctors usually observed that body temperature decreased, in some cases only a little alteration remained in the evening hours. SpO${}_{2}$ gradually rose over the days, until it reached almost normal values (from 90% to 96–98%) and HR gradually normalised. The gradual improvement of vital parameters underlined the good response of patients to the anti-Covid drug therapy. Thanks to the possibility of doing extra-measurements, the doctors were also able to verify the walking test, which consists in measuring SpO${}_{2}$ before and after 6 minutes of walking. Together with the stabilisation of vital parameters, the walking test was usually passed (i.e., SpO${}_{2}$ did not decrease at the end of the test).

**Figure 2:**
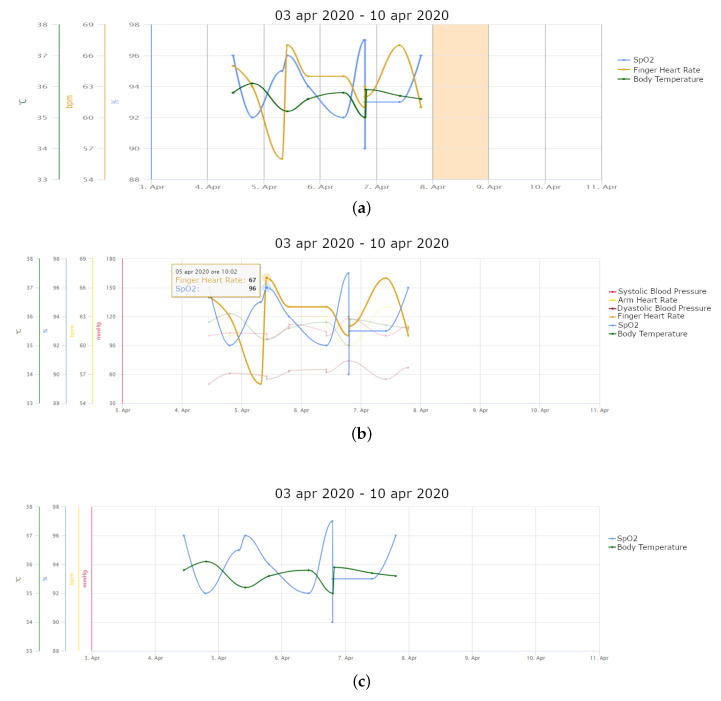
Trend of measurements of a patient aged 71 years with atrial fibrillation: (**a**) SpO${}_{2}$, finger HR and body temperature; (**b**) evidence of a measurement of SpO${}_{2}$ and finger HR; (**c**) highlighting of SpO${}_{2}$ and body temperature.

Figure 2a shows an example of a vital parameter (SpO${}_{2}$, finger HR and body temperature) graph in the web platform, while Figure 2b highlights a measurement of SpO${}_{2}$ and finger HR on 5 April 2020. The patient, a female aged 71 years with atrial fibrillation, is one of the first patients enrolled during the first wave and represents an example of a good clinical response with drug therapy. She was recruited the tenth day from the first symptoms (fever, myalgia, headache and dry cough), even though she had not yet taken a molecular swab, as her condition was critical. However, the care team decided to treat her at home avoiding hospitalisation thanks to the availability of the telemedicine platform. From the detail of SpO${}_{2}$ and body temperature (Figure 2c), it is possible to note that the patient executed a walking test on 6 April 2020 and failed it (SpO${}_{2}$ decreased from 97% to 90%). However, SpO${}_{2}$ reached almost good values in normal situations during the days. On the contrary, body temperature was already below 37.5 °C at the time of enrolment and has never risen. The sensors were used also to monitor her husband at the onset of the first symptoms. He entered his measurements manually in the web platform, exploiting the new functionality of the system. Both they overcame the disease without hospitalisation, helped by the continuous monitoring provided by Tel.Te.Covid19.

Because of the good results achieved with the monitoring protocol adopted during the first wave, the latter has become a model of enrolment in the entire Local Health Unit of North-West Tuscany during the current second wave, considering that timing is crucial to avoid hospital admissions: patients in the first stage should be monitored with the moderate monitoring plan with their own sensors and manually enter their vital parameters in the web platform, while patients in the early second stage, after a visit of the GP on average between the sixth and the tenth day from the first symptoms, should be given a home kit and monitored with the intensive monitoring plan.

Moreover, the ability of the doctor to guarantee the continuity of care was formalised after the first wave in Tuscany, with the institution of USCA doctors for the management of Covid-19 patients. They are in charge of following the infected patients and eventually enrolling them in Tel.Te.Covid19 according to the stage of their disease and to the developed model. At the moment of recruitment of a patient in the telemedicine system, the USCA team becomes part of the care team together with his/her GP. In this way, they share electronic medical records and are able to reproduce the treatment methods typical of a hospital admission at home, with possible rapid change of the therapy if not effective.

**Figure 3:**
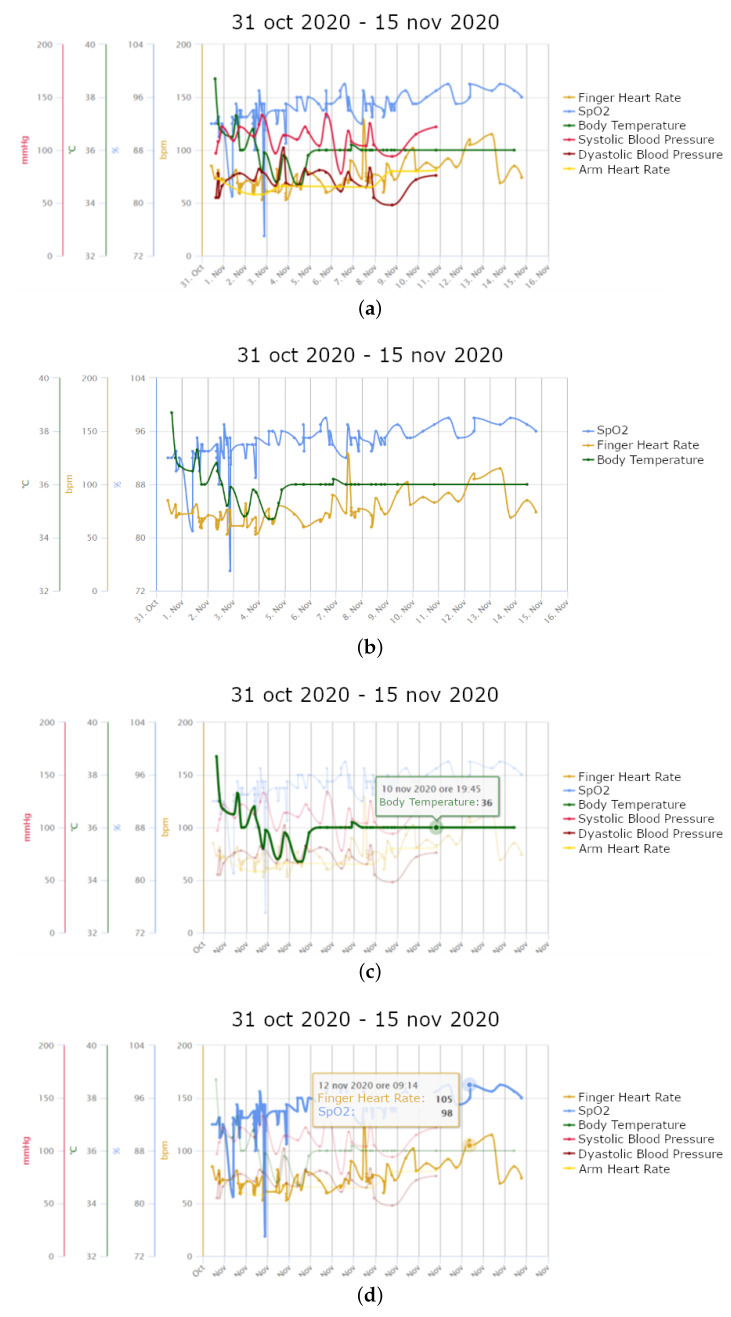
Trend of measurements of a patient aged 51 years without comorbidities: (**a**) all vital parameters; (**b**) SpO${}_{2}$, finger HR and body temperature; (**c**) highlighting of body temperature; (**d**) highlighting of SpO${}_{2}$ and finger HR.

Figure 3a,b show two vital parameter graphs (all vital parameters and only SpO${}_{2}$, finger HR and body temperature selected, respectively) of a male patient aged 51 years without comorbidities, enrolled by USCA doctors with intensive monitoring plan after 9 days from the first symptoms (fever, myalgia, headache, dry cough and right chest pain) on 31 October 2020. A visit of his GP showed bilateral pneumonia. The continuous monitoring provided with Tel.Te.Covid19 avoided a hospital admission that would have been certain without the telemedicine platform due to his condition. Figure 3c shows the gradual decrease of fever, which stabilised after three days at around 36 °C. Also SpO${}_{2}$ gradually normalised (Figure 3d), reaching values around 100% after a few days. A walking test was not passed on 3 November 2020 (SpO${}_{2}$ reduced from 95% to 88%), while it was passed on 8 November 2020 (SpO${}_{2}$ was 96% and 95%, at the beginning and at the end of the test, respectively), showing great improvement in lung capacity. This patient is only another example of a good clinical response to Covid-19 drug therapy, which can be monitored and verified in such detail with the help of the telemedicine platform.

Starting from March 2020, in the Local Health Unit of North-West Tuscany 40 GPs and USCA doctors and about 180 symptomatic patients have been involved in Tel.Te.Covid19 with intensive monitoring.

The enrolment model developed and the opportunities given by the telemedicine platform led to no hospitalisations and no deaths among the monitored patients, thanks to the great timing of recruitment (around the tenth day from the first symptoms for intensive monitoring), the continuous remote monitoring and the consequent possibility to rapidly change the drug therapy if necessary. Then, Tel.Te.Covid19 have contributed to the reduction of the impact on the National Health System and to the better allocation of the healthcare resources, thanks to a minor number of hospital admissions.

In addition, in the “classical” management of the disease, at least one visit per day by the GP is required to monitor the evolution of pulmonary involvement of patients with symptoms, and vital parameters are monitored via phone calls, wasting a lot of time. On the contrary, with Tel.Te.Covid19 the visits become two in the entire period of the disease: the first one at the time of enrolment in the intensive phase, to confirm the transition to the second stage of Covid-19; the second one to verify the pulmonary recovery and to stop or reduce (from intensive to moderate) the remote monitoring. In fact, the effectiveness of the prescribed drug therapy and the evolution of the vital parameters can be verified remotely. The reduction of the contacts for the visits reduces also the risk of infection for the doctors themselves: in the last year no GP and no USCA doctor using Tel.Te.Covid19 has been infected. Phone calls have been replaced by automatic collection of vital parameters, while video calls added to the platform have helped for some particular checks related to the vital parameters graph.

All patients involved in Tel.Te.Covid19 have given a positive feedback to the platform. Patients with advanced clinical syndrome and significant lung involvement have gained great reassurance from intensive monitoring of their vital parameters, thanks to the fact that they have been continuously followed by the doctors also without visits and phone calls. The video call feature has been really appreciated, because patients do not feel alone and left to themselves. The GUI of both the mobile application and the web platform is considered easy-to-use. Patients, including the elderly, had no difficulty in performing self-measurements required by the monitoring plan, because of the great usability of the system. In fact, the GUI and the entire platform were inherited from an existing telemedicine platform (Tel.Te.C.) designed for chronic patients, usually elderly, with a simple human-machine interface and visual technical explanations for the use of the sensors. Particularly, if the patient is not willing to perform an extra-measurement, there is no need to touch the application: the patient has just to see the reminder and follow the graphical instructions to wear and activate the sensor. Moreover, the number of measurements, especially in the first days of using the platform, was greater than requested, showing excellent compliance. Also the family members of enrolled patients have been able to use the provided sensors and enter the measurements manually on the web platform, even if this process is a little bit more difficult than the automatic acquisition via tablet.

On their side, GPs were able to follow the trend of the various vital parameters over time, highlighting the patients’ response to the current therapeutic protocol and the possible onset of side effects, allowing them to change therapy in time. Their comments have always been positive, since Tel.Te.Covid19 has really simplified the management of Covid-19 patients for them.

Thanks to the good results achieved on the territory with Tel.Te.Covid19, the GPs already involved in the project are continuing to enrol patients, and some other Tuscan GPs are approaching the platform, thanks to the great usability, as well as usefulness, of the Tel.Te.Covid19 platform, which presents the graphs of the vital parameters in an easy way in the web platform, is really easy-to-use for the patients (with and without the tablet), and warns the doctors if some of their patients’ parameters are out of threshold. While using the platform, GPs are making their own requests to upgrade the system and increase support for their work. In particular, the next step will be integration with intelligent, expert algorithms, which will provide them with greater support in enrolling patients and setting up personalised clinical monitoring plans.

## 4. Conclusions

With the arrival of the Covid-19 pandemic, in Tuscany (Central Italy), an existing telemedicine system, already used to remotely monitor chronic patients, has been updated with new functionalities to respond to management problems of infected and high-risk patients, creating the Tel.Te.Covid19 platform. This system is composed of a home monitoring kit, to be delivered directly to the patients, a professional kit, for nurses and healthcare operators to manage multiple patients, and a web platform, where the doctors can monitor the trend of the vital parameters measured at home. The vital parameters that can be monitored are body temperature, oxygen saturation, heart rate, blood pressure and weight. The web platform has been improved to allow patients without Bluetooth sensors to enter their measurements manually and to allow entire families to use the same home kit, increasing the scalability and flexibility of the system. Moreover, video calls have been added, giving the doctors the possibility to do in-app calls and see the patients remotely. We have provided also two standard monitoring plans (moderate and intensive), speeding up the moment of enrolment.

Tel.Te.Covid19 has been used by 40 general practitioners and about 180 patients since March 2020. In particular, patients with severe symptoms (second stage of the disease) have been enrolled. The system has achieved positive results: no hospitalisations, no deaths, reassurance for patients and positive feedback from both patients and doctors. In fact, the platform has a great usability and usefulness, since it presents the graphs of vital parameters in a simple way, raises alarms if parameters are out of range, reminds the user to collect measurements and has an easy graphical user interface (both on mobile and on web).

## Figures and Tables

**Table 1:** Bluetooth sensors provided in the home kit of Tel.Te.Covid19.

Vital Parameter	Device
Temperature	TAIDOC TD-1241B [22]
SpO${}_{2}$ and finger HR	GIMA OXY 10 [23]
Blood pressure	AND UA-767PBT-Ci [24]
Weight	AND UC-351PBT-Ci [25]

## Data Availability

The data presented in this study are available on request from the corresponding author. The data are not publicly available due to privacy.
